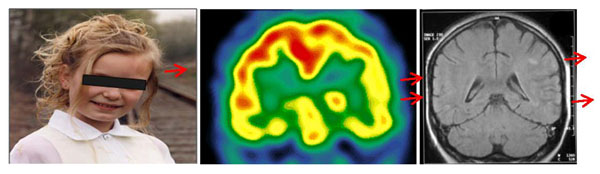# Linear scleroderma en coup the sabre, progressive facial hemiatrophy and Rasmussen encephalitis : a single disease spectrum?

**DOI:** 10.1186/1546-0096-9-S1-P79

**Published:** 2011-09-14

**Authors:** M Morren, C Despontin, C Wouters

**Affiliations:** 1 Dept of Pediatric Dermatology, Leuven University Hospital, Belgium; 2 Dept of Pediatric Dermatology, Children’s Hospital Luxembourg; 3 Pediatric Rheumatology, Leuven University Hospital, Belgium

## Background

Progressive hemifacial atrophy (PFH) and linear scleroderma en coup de sabre (LSCS) may be accompanied by neurologic symptoms and other extra-cutaneous manifestations.

## Aim

To investigate and compare clinical/immune characteristics of patients with LSCS and PFH.

## Methods

Retrospective study of 12 patients presenting at 2 pediatric dermatology clinics with linear scleroderma and/or hemifacial atrophy

## Results

9 patients presented with LSCS, followed in 5 of them by progressive hemifacial atrophy (PFH) within 2 years. 3 patients presented with PFH, all 3 had additional scleroderma lesions. Their median(range) age at presentation was 8(4-17)yrs.

Extracutaneous manifestations were equally found in LSCS and PFH+LSCS patients. They comprised asymmetry of tooth arches/missing teeth(1), ophthalmologic problems (eyelid ptosis, enophtalmia, bilateral anterior uveitis and renal papillary asymmetry)(4), epileptic seizures with hyperintense signals on MRI and hypoperfusion on SPECT in 1 LSCS (fig) and 1 PFH patient, severe migraine attacks(2), polyarthritis(2), vitiligo(1), celiac disease(1).

ANA were found in 3 LSCS, 2 PFH+LSCS patients. Distinct oligoclonal IgG bands were found in CSF (not in blood) in patients with PFH and seizures. A skin biopsy in a PFH lesion showed fibrosis associated with a lymphocytic infiltrate, IgM and IgG deposits. A brain biopsy in one PFH patient with epilepsy was consistent with Rasmussen encephalitis.

In patients with severe skin +- neurological lesions, treatment with steroids/high-dose MTX resulted in improvement/stabilization of clinical and MRI abnormalities

## Conclusion

Our case series endorses the concept of a single disease spectrum encompassing LSCS and PFH, with a common immune-inflammatory pathogenesis. A possible relationship with RE is suggested as well.

**Figure 1:**